# P09 Uncommon cause of back pain

**DOI:** 10.1093/rap/rkab068.008

**Published:** 2021-10-19

**Authors:** Shilpa Jagadeesh, Elizabeth Fiona Wood

**Affiliations:** Furness General Hospital, Barrow-in-Furness, United Kingdom

## Abstract

**Case report - Introduction:**

Back pain is one of the common musculoskeletal presentations to GPs and rheumatologists. While common causes are frequent, our patient required extensive investigation to reach a clinical diagnosis. This case draws attention to complexities involved and aims to refresh knowledge of less common causes.

**Case report - Case description:**

A 40-year-old man presented to the GP with a few months history of gradual onset worsening low back and buttock pain, which was non-radiating resulting in reduced mobility. There was no preceding illness, systemic symptoms, gross neurology or associated features.

Bloods revealed raised inflammatory markers and XR-pelvis showed lytic lesion left ischium. MRI showed multiple destructive lesions in lumbar spine and sacrum, suggestive of malignancy.

Gentleman was referred for ambulatory care assessment as unable to work, using a wheelchair and requiring significant analgesia.

CT TAP indicated left hip synovial thickening possibly inflammatory arthritis, confirmed lytic bony lesions but found no primary.

He was referred for CT-guided bone biopsy and cancer of unknown primary MDT. Tumour markers were tested and symptom management assistance was sought from pain team and palliative care. Orthopaedic colleagues reviewed and discharged for management by palliative care team.

He was discussed with rheumatology. Detailed review revealed tuberculosis (TB)-related death in the family. His IGRA returned positive. There was vague history of fluctuating weight over the few months before presentation to GP. He was of Filipino origin settled in UK for over 10 years and no foreign travel for last few years. PET-CT showed active lytic bony lesions. MRI hip suggested likelihood of tuberculosis due to synovial thickening with septation. Hip aspiration for culture couldn’t be performed as effusion resolved by then.

Bone biopsy showed granulomatous inflammation with plasma cells, staining and culture were negative for acid fast bacilli. Specialist opinion on biopsy specimen was sought which ruled out Langerhans cell histiocytosis and haematological malignancy including multiple myeloma.

Serum ACE, ANCA, ANA were all negative and C4 mildly raised, C3 normal. Immunoglobulin electrophoresis didn't show monoclonal bands.

Anti-TB chemotherapy was commenced based on probability and patient recovered almost back to baseline in 8 months. Repeat MRI after 4 months of treatment showed no interval change.

**Case report - Discussion:**

Extrapulmonary TB accounts for 3% of all TB and skeletal TB 10% of extrapulmonary TB. Skeletal TB presents with minimal systemic symptoms. Drug resistance is emerging around the world due to incomplete treatment and migration from countries with high prevalence of MDR-TB.

Common differentials for lytic bone lesion include secondary or primary malignancy and multiple myeloma/plasma cell tumours. Biopsy is gold standard for diagnosis. The differential diagnosis for bone marrow granuloma is wide but TB, sarcoid and malignancy remain common considerations. It is rare for sarcoidosis to present with isolated bone marrow granuloma and usually there are systemic symptoms. Infectious causes of bone marrow granuloma include brucellosis, typhoid, Q fever, histoplasmosis, Epstein Barr virus and cytomegalovirus but usually present with significant systemic symptoms. In addition to routine tests and biopsy for TB, adenosine deaminase assay from cerebrospinal fluid could be used to aid diagnosis in cases where it would alter management (as per NICE guidance).

Challenges exist from the patient perspective. This young man had constantly changing diagnostic and prognostic considerations which he found confusing. Culture did not provide a confirmation of diagnosis so he found it hard to be sure. He was off work in a wheelchair on palliative care for suspected malignancy early in the year. In the mid part of year he was told it was bone TB and commenced treatment. In the later part of year he was walking unaided and able to reduce pain medication.

Challenges also exist from the rheumatologist’s perspective. This was a young man with minimal systemic symptoms on a palliative care pathway. There was constantly evolving change in management and multispecialty involvement with no ownership. Locally TB is managed by the respiratory team; however, they felt it more appropriate to offer advice regarding treatment and for this to be managed by rheumatology. TB nurses for the region did provide assistance for contact tracing and notification to public health.

**Case report - Key learning points:**

Consider TB as differential diagnosis of lytic bone lesion and/or bone marrow granuloma.

Bone and spine TB present with no or minimal systemic symptoms. Family and immigration history may be important.

TB blood tests (IGRA) preferred over skin tests in BCG vaccinated as per CDC, USA.

Bone marrow granuloma in TB is noncaseating mainly and caseating in 29% as per a study.

Presence of acid-fast bacilli in TB bone marrow granuloma is rare and culture is positive in only 15% of cases as per a case series.

PET-CT cannot distinguish between TB, malignancy and inflammatory lesions but can be used to assess improvement after therapy.

MRI may show resolution of bony changes in bone TB 9 months post treatment. This would be an appropriate time to consider repeat imaging.

NICE recommends 12 months of treatment for disseminated TB or spine TB with central nervous system involvement but commonest duration in literature is 18 months for bone TB. NICE recommends 6 months anti-TB treatment for spinal tuberculosis without central nervous system involvement. There is no need to routinely extend treatment beyond these for people with HIV and active TB.

Comorbidities that may need working with multidisciplinary specialist teams include HIV, severe liver disease, stage 4 or 5 chronic kidney disease, eye disease/impaired vision, pregnancy/breastfeeding, history of alcohol/substance misuse.

Referral of patients with spinal TB for surgery to be considered as per NICE for patients with spine instability or cord compression.

Future consideration for monitoring of anti-TB drug therapy – drug-induced liver injury from anti-TB therapy was found to be 8.3% in a north-west London study and authors suggested routine 2-week check of liver function after commencing treatment (>30 u/l rise in pretreatment ALT predicted risk of drug-induced liver injury).

